# Endoscopic detection and diagnosis of gastric cancer using image‐enhanced endoscopy: A systematic review and meta‐analysis

**DOI:** 10.1002/deo2.418

**Published:** 2024-08-13

**Authors:** Osamu Dohi, Mayuko Seya, Naoto Iwai, Tomoko Ochiai, Junki Yumoto, Hiroki Mukai, Katsuma Yamauchi, Reo Kobayashi, Ryohei Hirose, Ken Inoue, Naohisa Yoshida, Hideyuki Konishi, Yoshito Itoh

**Affiliations:** ^1^ Molecular Gastroenterology and Hepatology Graduate School of Medicine, Kyoto Prefectural University of Medicine Kyoto Japan; ^2^ Department of Infectious Diseases Graduate School of Medical Science, Kyoto, Prefectural University of Medicine Kyoto Japan

**Keywords:** blue laser imaging, gastric cancer, image‐enhanced endoscopy, linked color imaging, narrow‐band imaging

## Abstract

**Objectives:**

We aimed to conduct a systematic review and meta‐analysis to assess the value of image‐enhanced endoscopy including blue laser imaging (BLI), linked color imaging, narrow‐band imaging (NBI), and texture and color enhancement imaging to detect and diagnose gastric cancer (GC) compared to that of white‐light imaging (WLI).

**Methods:**

Studies meeting the inclusion criteria were identified through PubMed, Cochrane Library, and Japan Medical Abstracts Society databases searches. The pooled risk ratio for dichotomous variables was calculated using the random‐effects model to assess the GC detection between WLI and image‐enhanced endoscopy. A random‐effects model was used to calculate the overall diagnostic performance of WLI and magnifying image‐enhanced endoscopy for GC.

**Results:**

Sixteen studies met the inclusion criteria. The detection rate of GC was significantly improved in linked color imaging compared with that in WLI (risk ratio, 2.20; 95% confidence interval [CI], 1.39–3.25; *p* < 0.01) with mild heterogeneity. Magnifying endoscopy with NBI (ME‐NBI) obtained a pooled sensitivity, specificity, and area under the summary receiver operating curve of 0.84 (95 % CI, 0.80–0.88), 0.96 (95 % CI, 0.94–0.97), and 0.92, respectively. Similarly, ME‐BLI showed a pooled sensitivity, specificity, and area under the curve of 0.81 (95 % CI, 0.77–0.85), 0.85 (95 % CI, 0.82–0.88), and 0.95, respectively. The diagnostic efficacy of ME‐NBI/BLI for GC was evidently high compared to that of WLI, However, significant heterogeneity among the NBI studies still existed.

**Conclusions:**

Our meta‐analysis showed a high detection rate for linked color imaging and a high diagnostic performance of ME‐NBI/BLI for GC compared to that with WLI.

## INTRODUCTION

Gastric cancer (GC) is the fifth most common cancer in global cancer and fourth in mortality, estimated to account for approximately 6.8% of all cancer‐related deaths by 2022.^1^ When curative resection is achieved through endoscopic resection, early GC can attain a 5‐year survival rate of over 90 %.^2^ Therefore, early detection of GC via esophagogastroduodenoscopy (EGD) is important to reduce GC mortality.

White‐light imaging (WLI) is widely used to detect and characterize GC during EGD. However, GC is missed in 4.6–25.8% of patients when using WLI.^3^ Moreover, the diagnoses of premalignant gastric lesions and GC have shown an inadequate correlation between WLI and histology.^4^ Thus, the detectability and diagnostic accuracy of WLI alone are not sufficient to prevent missed GCs and incorrect GC diagnoses.

In recent years, several image‐enhanced endoscopy (IEE) techniques, including blue laser imaging (BLI), linked color imaging (LCI), narrow‐band imaging (NBI), and texture and color enhancement imaging (TXI), have been developed to enhance marginal differences in mucosal color and structure with white light or short‐wavelength light sources. Magnifying endoscopy with NBI (ME‐NBI) is a useful diagnostic tool for GC in Japan.^5^ ME‐NBI also demonstrates a high concordance with gastric histology, including gastric intestinal metaplasia and dysplasia, and is superior to WLI in Western countries.^6^

However, the effectiveness of these IEE systems for detecting and diagnosing GC has not yet been systematically evaluated. Therefore, in this study, we conducted a systematic review and meta‐analysis to assess the efficacy of IEEs in the detection and diagnosis of GC compared to that of WLI.

## METHODS

### Protocol

This systematic review and meta‐analysis was performed according to the guidelines set forth by the Preferred Reporting Items for Systematic Reviews and Meta‐Analyses Statement (Table S1).

### Search strategy

Relevant studies were identified by searching the PubMed, Cochrane Library, and Japan Medical Abstracts Society databases up to December 2023. Articles were identified for studies including on the detection and diagnostic performance between IEE and WLI for GC or high‐grade intraepithelial neoplasia using the following search keywords: (“white light” OR “WLI” OR “WLE” OR “image‐enhanced endoscopy” OR “IEE” OR “narrow band” OR “narrow band imaging” OR “NBI” OR “BLI” OR“ blue laser imaging” OR “LCI” OR “linked color imaging” OR “texture and color enhancement imaging” OR “TXI”) AND (“gastric dysplasia” OR “gastric high‐grade dysplasia” OR “stomach neoplasm” OR “gastric neoplasm” OR “stomach cancer” OR “gastric cancer”) AND (“detection” OR “detectability” OR “diagnosis” OR “diagnostic accuracy” OR “diagnostic performance” OR “sensitivity” OR “specificity”).

### Study selection

After removing duplicates and overlapping publications, two investigators (Osamu Dohi and Mayuko Seya) performed an initial screening of the identified abstracts or titles to determine articles eligible for further review. The second screening was based on a full‐text review of studies that met the inclusion criteria. The same two investigators independently assessed the full text for eligibility; discrepancies were resolved via consensus or were determined by a third investigator (Naoto Iwai). Moreover, the references of the included articles were manually reviewed to identify additional potentially relevant studies.

### Inclusion and exclusion criteria

Inclusion criteria were original articles with primary or secondary outcomes regarding the detection, accuracy, sensitivity, or specificity of WLI and IEE/ME‐IEE for GC. The exclusion criteria were as follows: case reports, meta‐analyses, reviews, letters, comments, congress abstracts, guidelines, animal studies, studies with fewer than 10 cases, studies published in languages other than English, studies with non‐pathological data, and studies with unavailable statistical data for true positives, true negatives, false positives, and false negatives.

### Data extraction

The following data were extracted by Osamu Dohi and independently checked by Mayuko Seya: name of first author, year of publication, country, study period, study design, number of patients and lesions included, participant characteristics (number of lesions/areas biopsied; age; sex), IEE technology assessed (BLI, LCI, and NBI), outcome (GC), basis of analysis (per‐patient and per‐lesion), and measuring diagnostic performance (sensitivity, specificity, and odds ratio [OR]). The reference standard was the histopathology of specimens obtained from biopsy, endoscopic resection, or surgical resection.

### Outcome measurement

The detection rate and diagnostic performance of WLI and each IEE technology for GC were analyzed individually, considering per‐patient or per‐biopsy analyses. Risk ratios (RRs) with 95% confidence intervals (CIs) were used for GC detection in the investigated trials. The diagnostic accuracy measures were derived from a 2 × 2 contingency table for each trial. Finally, the pooled measures (sensitivity, specificity, and diagnostic OR [DOR]) with their respective 95% CIs, and summary receiver operating curves were calculated.

### Quality assessment

The Cochrane Collaboration tool for randomized trials (RoB2) was used to assess the risk of bias among studies of GC detection.^7^ The Quality Assessment of Diagnostic Accuracy Studies, second version (QUADAS‐2) was used to assess the quality of diagnostic accuracy studies. The quality assessment was independently performed by two authors (Osamu Dohi and Naoto Iwai), and eventual disagreements were discussed with a third author (Mayuko Seya). Publication bias was assessed using funnel plots and Egger's linear regression test when five or more studies were eligible for inclusion.

### Statistical analysis

Pooled estimates, their corresponding 95% CIs, and *p*‐values for dichotomous variables were calculated using the random‐effects model in the meta‐analysis. The DOR of a test is defined as the ratio of the odds of positivity in the disease group relative to the odds of positivity in the non‐diseased group. This relationship is expressed as follows:

$$\begin{array}{ccc} \mathrm{DOR} & = & \frac{\mathrm{True}\;\mathrm{positive}/\mathrm{False}\;\mathrm{negative}}{\mathrm{False}\;\mathrm{positive}/\mathrm{True}\;\mathrm{negative}}\\ & & =\frac{\mathrm{Sensitivity}/\left(1-\mathrm{Sensitivity}\right)}{\left(1-\mathrm{Specificity}\right)/\mathrm{Specificity}} \end{array}$$

Heterogeneity among studies was evaluated using Spearman's rank correlation coefficient and the *I* ^2^ statistic. Spearman's rank correlation coefficient was employed to explore potential threshold effects between sensitivity and specificity within the primary studies included in this meta‐analysis. *I* ^2^ values of 0–30%, 30–60%, 60–75%, and more than 75% represented no, mild, moderate, and high heterogeneity, respectively.^8^ We judged inconsistencies in the results between studies as indicating heterogeneity in the Cochrane Q test if the *p*‐value was < 0.05. Statistical significance was set at *p* < 0.05. All data analyses were conducted using EZR version 4.2.2 (Saitama Medical Center, JichiMedical University) and MetaDiSc 1.4 software.

## RESULTS

### Study selection

**FIGURE 1:**
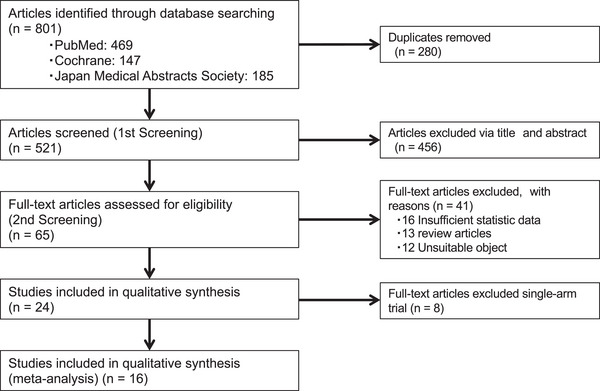
Flow diagram for assessment of studies.

A total of 801 studies were identified using the three databases, and 280 duplicates were removed. The screening and evaluation of titles and abstracts led to the exclusion of 456 studies. The full‐text details of the remaining 65 studies were assessed, and 41 were excluded. Of the 24 studies, eight were excluded because they included the outcomes of only IEE or WLI with IEE. Finally, 16 comparative studies between WLI and IEE, including five for GC detection and 11 for diagnostic performance, were analyzed in this systematic review and meta‐analysis (Figure 1).^9^, ^10^, ^11^, ^12^, ^13^, ^14^, ^15^, ^16^, ^17^, ^18^, ^19^, ^20^, ^21^, ^22^, ^23^, ^24^

### Characteristics of studies

Table 1 presents the characteristics of the studies included in the qualitative synthesis of the meta‐analyses. Of the five randomized controlled trials comparing WLI and IEEs for GC detection, two studies used NBI,^9^, ^11^ one used BLI‐bright,^10^ and the other two utilized LCI.^12^, ^13^ In terms of the design, four studies used a crossover design,^10^, ^11^, ^12^, ^13^ and one employed a parallel design.^9^

**TABLE 1:** Characteristics of randomized controlled trials investigating white‐light imaging (WLI) versus image‐enhanced endoscopy for gastric cancer detection.

					No. of patients		No. of GC		Detection rate, %	
Author	Country	Center	Study design	IEE system	WLI	IEE	WLI	IEE	WLI	IEE
Ang et al.^9^	East Asia	Multiple	Parallel	NBI	286	293	7	3	2.40	1.02
Dohi et al.^10^	Japan	Single	Crossover	BLI‐bright	298	298	12	26	4.03	8.72
Yoshida et al.^11^	Japan	Multiple	Crossover	NBI	2258	2265	44	53	1.90	2.30
Ono et al.^12^	Japan	Multiple	Crossover	LCI	752	750	20	37	2.66	4.93
Min et al.^13^	China	Multiple	Crossover	LCI	914	914	6	20	0.66	2.19

Abbreviations: BLI‐bright, blue laser imaging‐bright; GC, gastric cancer; IEE, image‐enhanced endoscopy; LCI, linked color imaging; NBI, narrow‐band imaging; WLI, white‐light imaging.

Table 2 shows diagnostic values of WLI and magnifying IEEs for GC among the studies in the qualitative synthesis of the meta‐analysis. Of the 11 studies comparing WLI and ME‐IEEs for diagnostic performance, eight utilized ME‐NBI,^14^, ^15^, ^16^, ^17^, ^18^, ^19^, ^20^, ^21^ and the other three used ME‐BLI.^22^, ^23^, ^24^ In terms of the study design, one randomized controlled trial,^16^ four prospective,^14^, ^15^, ^22^, ^24^ and six retrospective studies^17^, ^18^, ^19^, ^20^, ^21^, ^23^ have been reported. The years of publication or presentation were from 2015 to 2022. All the studies were conducted in Asia.

**TABLE 2:** Diagnostic values of white‐light imaging and magnifying image‐enhanced endoscopies for gastric cancer (GC).

									Diagnostic performance			
									WLI	M‐IEE	WLI	M‐IEE
Author	Country	Study Design	Diagnostic criteria	M‐IEE System	No. of patients	No. of lesions	No. of non‐GC lesions	No. of GC	Sensitivity	Specificity	Sensitivity	Specificity
Ezoe et al.^14^	Japan	Prospective	VSCS	NBI	53	57	27	30	0.230	0.670	0.700	0.890
Kato et al.^15^	Japan	Prospective	Original	NBI	111	201	187	14	0.429	0.610	0.857	0.914
Ezoe et al.^16^	Japan	RCT	VSCS	NBI	1356	353	156 (WLI) 157 (NBI)	20 (WLI) 20 (NBI)	0.400	0.679	0.600	0.943
Miwa et al.^17^	Japan	Retrospective	VSCS	NBI	135	135	79	56	0.696	0.57	0.875	0.974
Maki et al.^18^	Japan	Retrospective	VSCS	NBI	93	93	32	61	0.640	0.940	0.950	0.880
Tao et al.^19^	China	Retrospective	VSCS	NBI	508	643	619	24	0.750	0.895	0.917	0.995
Fujiwara et al.^20^	Japan	Retrospective	VSCS	NBI	99	103	71	32	0.437	0.816	0.780	0.929
Nonaka et al.^21^	Japan	Retrospective	Original	NBI	91	100	21	79	0.709	0.571	0.848	0.476
Dohi et al.^22^	Japan	Prospective	VSCS	BLI	530	127	95	32	0.469	0.800	0.938	0.916
Kitagawa et al.^23^	Japan	Retrospective	VSCS	BLI	93	100	45	55	0.591	0.604	0.791	0.722
Zhenming et al.^24^	China	Prospective	VSCS	BLI	235	249	224	25	0.720	0.953	0.92	0.981

Abbreviations: BLI, blue laser imaging; GC, gastric cancer; IEE, image‐enhanced endoscopy; LCI, linked color imaging; M‐IEE, magnifying image‐enhanced endoscopy; NBI, narrow‐band imaging; RCT, randomized controlled trial; VSCS, vessel and surface classification system; WLI, white‐light imaging.

### Detection rate of gastric cancer

**FIGURE 2:**
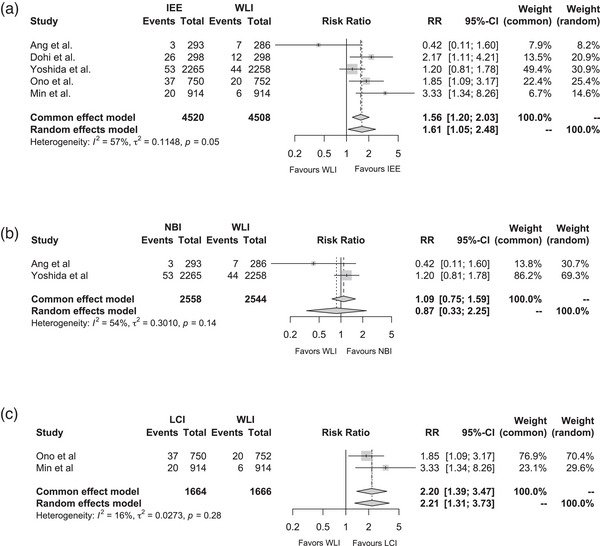
A quantitative analysis comparing IEE to WLI for the detection of gastric cancer (GC). (a) GC detection between IEE and WLI. (b) GC detection between NBI and WLI. (c) GC detection between LCI and WLI. CI, confidence interval; IEE, image‐enhanced endoscopy; LCI, linked color imaging; NBI, narrow‐band imaging; RR, risk ratio; WLI, white‐light imaging.

The detection rate was determined in five studies (*n* = 9028). In the LCI study,^12^ the number of detected GC cases was extracted from raw data obtained from the source. IEEs demonstrated a significant superiority over WLI (RR, 1.56; 95% CI, 1.20–2.03; *p* < 0.01) with mild heterogeneity (*I* ^2^ = 57%; Figure 2a). A relative funnel plot is shown in Figure S1. Egger's test indicated that publication bias was not statistically significant among the five studies (*p* = 0.92). Overall, two studies reported the detection rates of GC per patient using NBI and Olympus WLI (O‐WLI; *n* = 5102). The detection rate was not significantly higher in NBI than in O‐WLI (RR, 1.09; 95% CI, 0.75–1.59; *p* = 0.64) with mild heterogeneity (*I* ^2^ = 54%; Figure 2b). Additionally, two studies provided the detection rates of GC in LCI and Fujifilm WLI (F‐WLI; *n* = 3330), which was significantly improved in LCI compared with that in F‐WLI (RR, 2.20; 95% CI, 1.39–3.247; *p* < 0.01) with no heterogeneity (*I* ^2^ = 16%; Figure 2c).

For the four included studies, the risk of bias was judged as low in each domain, which is shown in the detailed risk assessment results (Table S2).

### Diagnostic performance for gastric cancer

In total, 11 studies included 3304 patients and 2161 lesions, including 448 cancerous and 1713 non‐cancerous lesions. In addition, eight studies assessed the diagnostic performance of O‐WLI and ME‐NBI,^14^, ^15^, ^16^, ^17^, ^18^, ^19^, ^20^, ^21^ and four evaluated that of F‐WLI and ME‐BLI for GC and non‐GC.^22^, ^23^, ^24^

**FIGURE 3:**
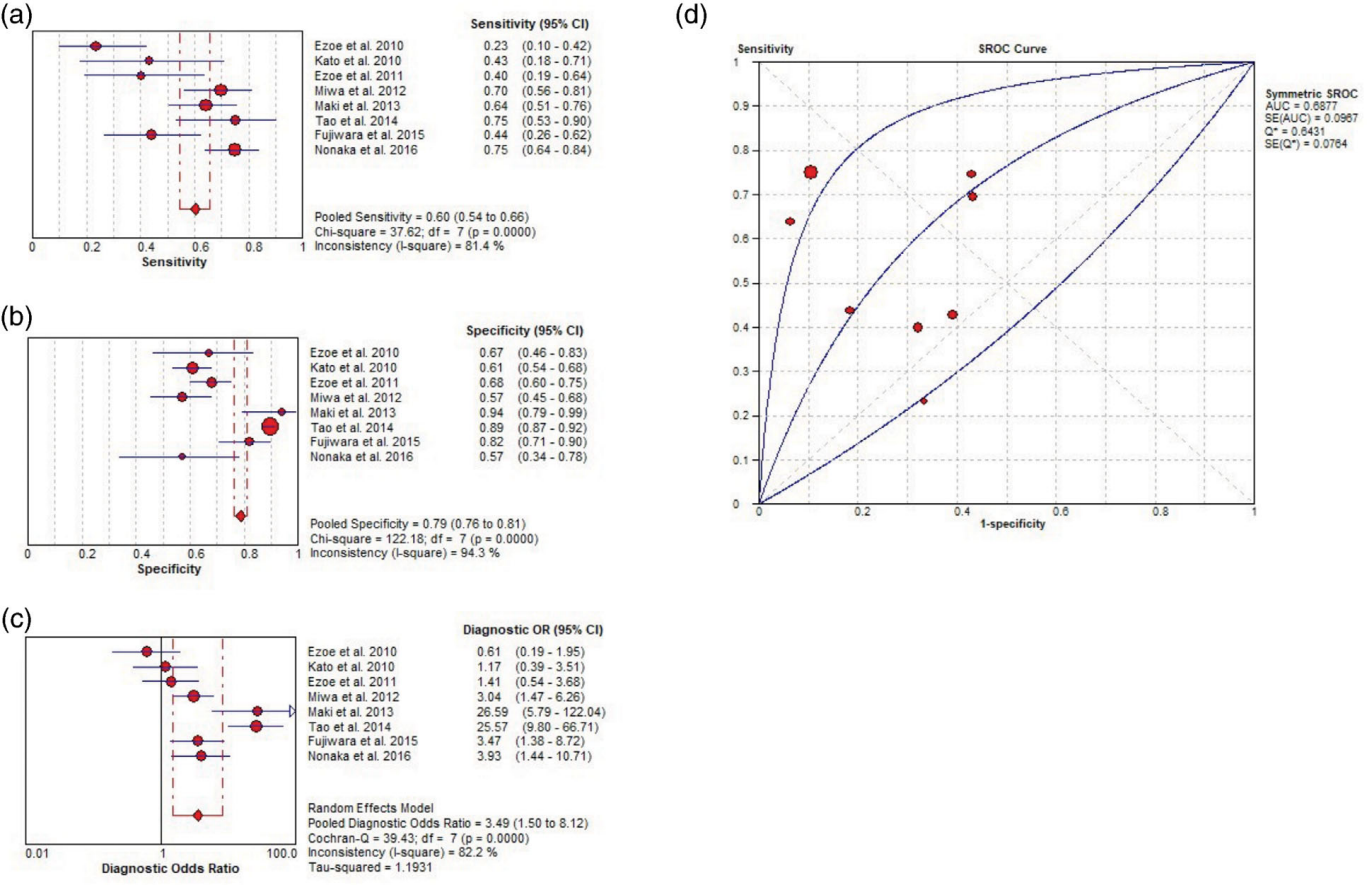
Per‐lesion analysis of the diagnostic performance of Olympus white‐light imaging (O‐WLI) for gastric cancer (GC). (a) Pooled sensitivity for O‐WLI to differentiate GC. (b) Pooled specificity for O‐WLI to differentiate GC. (c) Diagnostic odds ratio (OR) for O‐WLI to differentiate GC. (d) The summary receiver operating characteristic (SROC) curve for diagnosis using O‐WLI. AUC, area under the curve; CI, confidence interval; df, degrees of freedom; SE, standard error.

**FIGURE 4:**
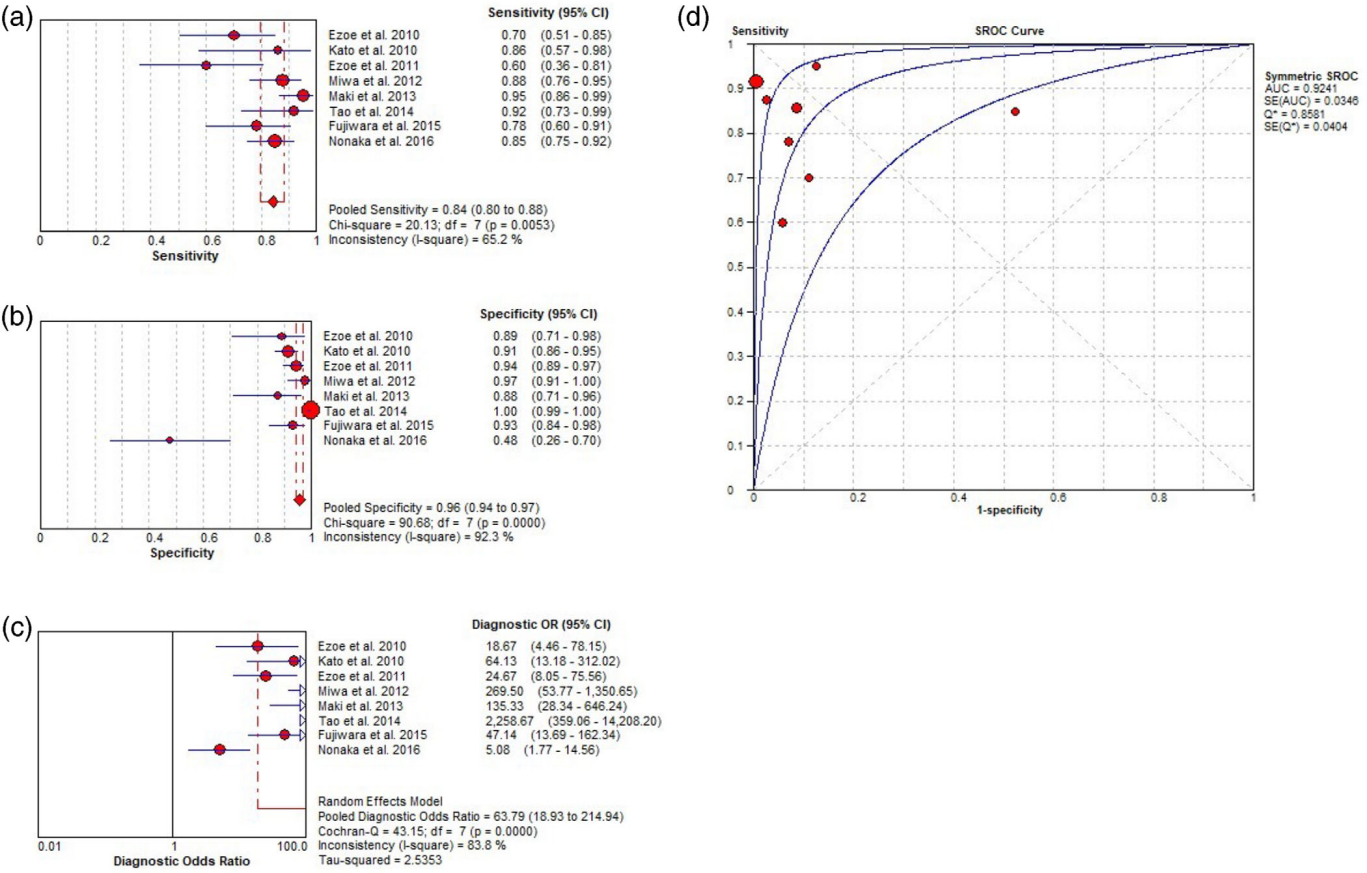
Per‐lesion analysis of the diagnostic performance of magnifying endoscopy with narrow‐band imaging (ME‐NBI) for gastric cancer (GC). (a) Pooled sensitivity for ME‐NBI to differentiate GC. (b) Pooled specificity for ME‐NBI to differentiate GC. (c) Diagnostic odds ratio (OR) for ME‐NBI to differentiate GC. (d) The summary receiver operating characteristic (SROC) curve for diagnosis using ME‐NBI. AUC, area under the curve; CI, confidence interval; df, degrees of freedom; SE, standard error.

Using a random‐effects model, the pooled sensitivity, pooled specificity, pooled DOR, and area under the summary receiver operating curve (AUC) of O‐WLI in the diagnosis of GC were 0.60 (95% CI, 0.54–0.66; *I* ^2^ = 81.4%), 0.79 (95% CI, 0.76–0.81; *I* ^2^ = 94.3%), 3.49 (95% CI, 1.50–8.12; *I* ^2^ = 82.2%), and 0.69 (standard error 0.10), respectively (Figure 3). Spearman correlation performed to assess the threshold effect showed a coefficient of –0.024 (*p* = 0.955), indicating a significant heterogeneity among the studies. Additionally, pooled sensitivity, pooled specificity, pooled DOR, and the AUC of ME‐NBI was 0.84 (95% CI, 0.80–0.88; *I* ^2^ = 65.2 %), 0.96 (95% CI, 0.94–0.97; *I* ^2^ = 92.3 %), 63.79 (95% CI, 18.93–214.94; *I* ^2^ = 83.3 %), and 0.92 (standard error 0.03), respectively (Figure 4). The Spearman correlation coefficient was –0.071 (*p* = 0.867), indicating large heterogeneity among the studies. These results showed that the diagnostic efficacy of ME‐NBI for GC was higher than that of WLI alone. However, there was still significant heterogeneity among the studies.

**FIGURE 5:**
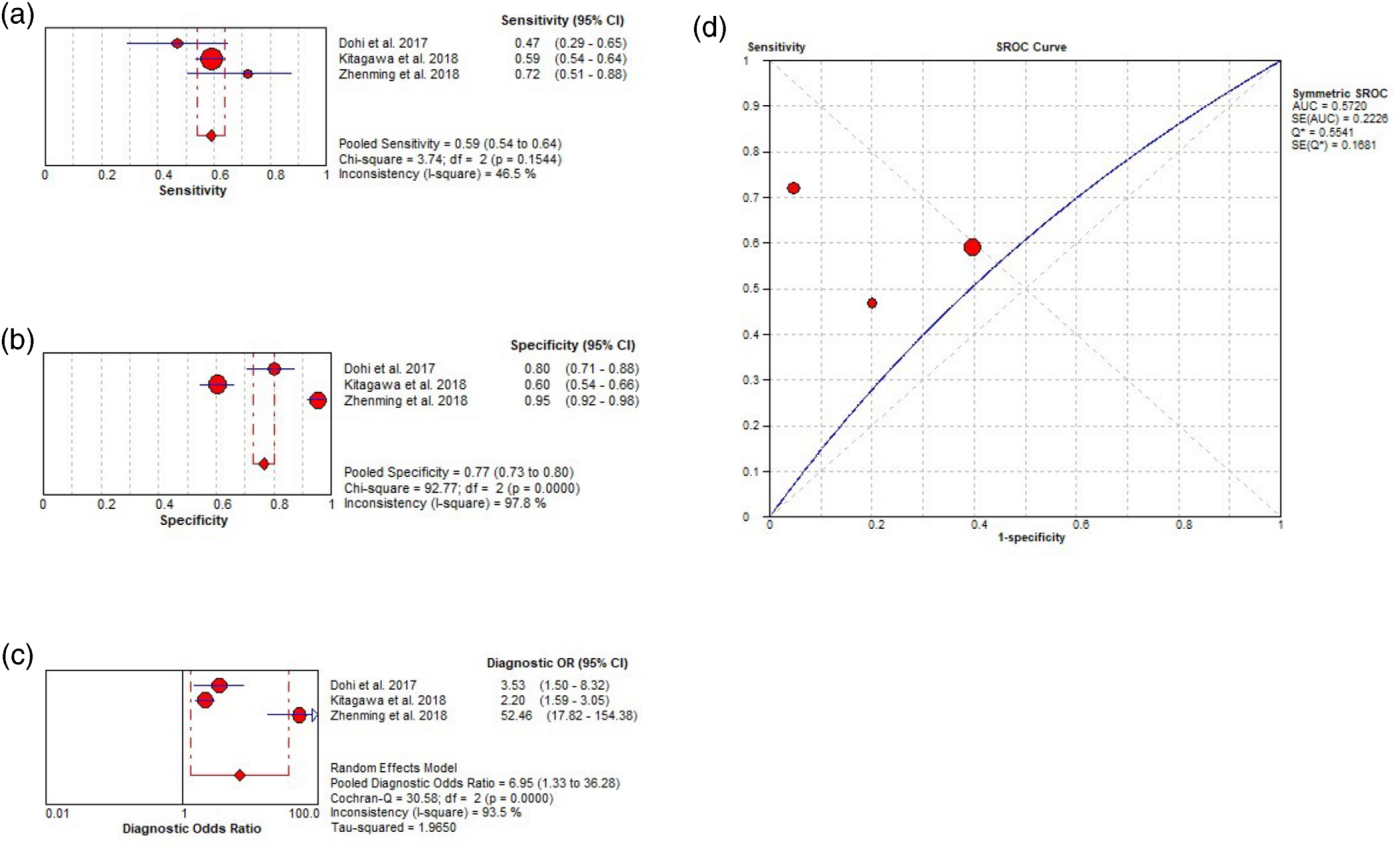
Per‐lesion analysis of the diagnostic performance of Fujifilm white‐light imaging (F‐WLI) for gastric cancer (GC). (a) Pooled sensitivity for F‐WLI to differentiate GC. (b) Pooled specificity for F‐WLI to differentiate GC. (c) Diagnostic odds ratio (OR) for F‐WLI to differentiate GC. (d) The summary receiver operating characteristic (SROC) curve for diagnosis using F‐WLI. AUC, area under the curve; CI, confidence interval; df, degrees of freedom; SE, standard error.

**FIGURE 6:**
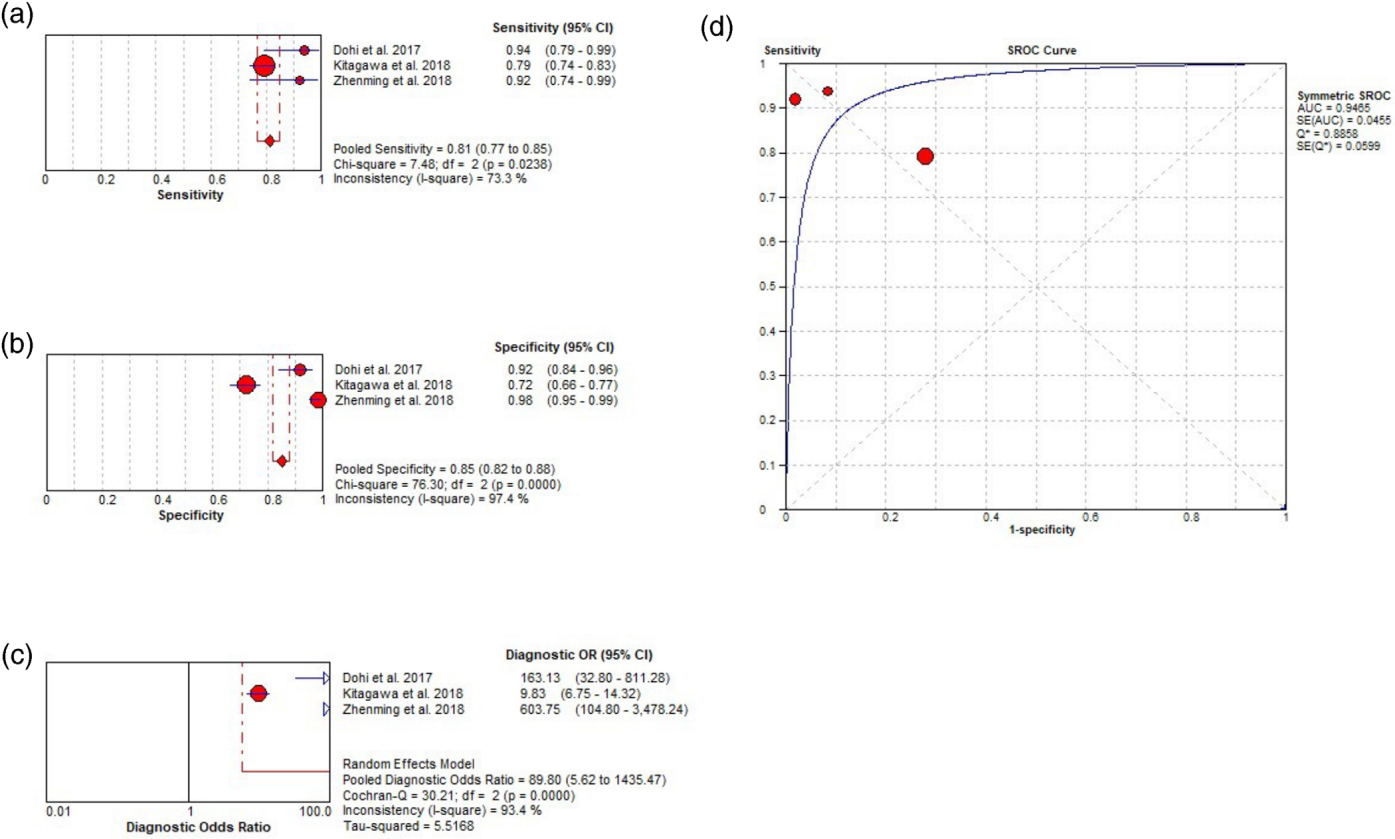
Per‐lesion analysis of the diagnostic performance of magnifying endoscopy with blue laser imaging (ME‐BLI) for gastric cancer (GC). (a) Pooled sensitivity for ME‐BLI to differentiate GC. (b) Pooled specificity for ME‐BLI to differentiate GC. (c) Diagnostic odds ratio (OR) for ME‐BLI to differentiate GC. (d) The summary receiver operating characteristic (SROC) curve for diagnosis using ME‐BLI. AUC, area under the curve; CI, confidence interval; df, degrees of freedom; SE, standard error.

The pooled sensitivity, pooled specificity, pooled DOR, and AUC of F‐WLI in the diagnosis of GC were 0.59 (95% CI, 0.54–0.64; *I* ^2^ = 46.5%), 0.77 (95% CI, 0.73–0.80; *I* ^2^ = 97.8%), 3.49 (95% CI, 1.50–8.12; *I* ^2^ = 82.2%), and 0.57 (standard error 0.22), respectively (Figure 5). Significant heterogeneity was observed among the studies; however, the results of the Spearman test revealed no threshold effect in these studies (r = –0.500, *p* = 0.667). Figure 6 shows that the pooled sensitivity, pooled specificity, pooled DOR, and AUC of ME‐BLI in the diagnosis of GC were 0.81 (95% CI, 0.77–0.85; *I* ^2^ = 73.3%), 0.85 (95% CI, 0.82–0.88; *I* ^2^ = 97.4%), 89.8 (95% CI, 5.62–1435.47; *I* ^2^ = 93.4%), and 0.95 (standard error 0.05), respectively. Although there was significant heterogeneity among the studies, the results of the Spearman test revealed no threshold effect in these studies (r = –0.500, *p* = 0.667). These results showed that the diagnostic efficacy of ME‐BLI was high and the stability of the results was good, with less heterogeneity among the studies.

The QUADAS‐2 assessment revealed that the studies exhibited a low‐to‐moderate risk of bias (Table S3 and Figure S2). All these studies utilized ME‐NBI or ME‐BLI and a diagnostic reference standard based on histopathology. WLI was used as a standard imaging modality in most studies. Patient selection was a primary source of bias due to non‐randomized patient assignment and the lack of exclusion criteria in some studies.

## DISCUSSION

To the best of our knowledge, this is the first meta‐analysis to compare the number of GC cases detected in patients using IEEs with WLI. Based only on high‐quality trials (five randomized controlled trials with more than 9000 patients), IEEs showed a significantly increased detection rate in GCs compared to WLI; moreover, LCI significantly increased the number of GC cases detected compared to that with WLI (OR: 2.20). No heterogeneity was observed among the studies employing LCI in terms of GC detection. Moreover, the rate of overlooked GC was lower when using LCI than when using WLI (1.1% and 2.3%, respectively).^13^ This may be because the color enhancement provided by LCI enables observation of the wide gastric lumen, allowing the endoscopists to identify slightly depressed or flat GCs compared to that of WLI.

In the present study, the detection rate of NBI was not significantly higher than that of O‐WLI. Therefore, non‐magnified NBI is not clinically superior to WLI for GC detection. Moreover, the rate of overlooked GC by second‐generation NBI is reported to be 22.4%, as detected using WLI.^11^ In the stomach, the distant images are still dark with the second‐generation NBI system because of the wide lumen, and it is often difficult to adequately observe the entire gastric lumen. Therefore, NBI should be used in combination with WLI, particularly for detecting GC. However, third‐generation brighter NBI has recently been developed. Therefore, it is anticipated that the third‐generation NBI may play a role in GC detection as it offers increased brightness. Regarding BLI‐bright, pooled analysis was impossible in a single study with a small number of participants.

This large‐scale meta‐analysis included 11 clinical studies on the application of both WLI and ME‐NBI/BLI for the diagnosis of GC. Our results showed that ME‐NBI and ME‐BLI had higher sensitivities and specificities than WLI did. Our findings align with those of most published studies that reported high diagnostic efficacy of vessel and surface classification system (VSCS), suggesting that VSCS is an efficient criterion for diagnosing GC. To date, VSCS utilizing ME‐NBI,^25^ which detects the presence of a lesion with a demarcation line, an irregular microsurface pattern, or an irregular microvessel pattern, is widely used as a diagnostic criterion for GC. BLI shares the physical principle of NBI, employing two laser lights to obtain the narrow‐band light. Consequently, BLI exhibits an equivalent diagnostic performance for diagnosing GC as NBI. However, there are large differences in the sensitivity in each NBI or BLI study, ranging from 0.60 to 0.95 and from 0.79 to 0.94, respectively.^14^, ^15^, ^16^, ^17^, ^18^, ^19^, ^20^, ^21^, ^22^, ^23^, ^24^ Therefore, these studies exhibit high heterogeneity in terms of the diagnostic performance for GC.

No reports have compared WLI between different endoscopic systems for the diagnosis and detection of GC. This meta‐analysis included different endoscopic systems with different light sources: xenon lamp for O‐WLI and laser for F‐WLI. The detection rate, sensitivity, and specificity of O‐WLI and F‐WLI for GC were similar, regardless of the light sources or endoscopic systems used.

Several meta‐analyses have been reported for GC detection and diagnosis using magnifying or image‐enhanced endoscopy.^26^, ^27^ For instance, Le et al. collected studies on GC detection using magnifying endoscopy^26^; however, they also included retrospective studies that aimed at GC diagnosis rather than GC detection. In another systematic review, Rodriguez‐Carrasco et al. primarily evaluated the diagnostic accuracy of ME‐NBI^27^; however, only a few included studies have compared WLI and ME‐NBI/ME‐BLI. Moreover, they included patients with known target neoplastic lesions, introducing a selection bias. Since a meta‐analysis requires the selection of appreciated studies, we focused on the selection of high‐quality reports for the purpose of meta‐analysis.

Some limitations of this meta‐analysis should be considered. First, most studies were conducted in countries at high risk for GC incidence, including East and Southeast Asia, mainly Japan. Consequently, our findings may not apply to low‐risk regions. Second, endoscopists were not blinded to each modality, which is common in most studies designed to assess different endoscopic devices. Third, there were fewer prospective evaluations and high heterogeneity among the studies regarding ME‐NBI/BLI diagnosis of GC.

In conclusion, our meta‐analysis showed a high detection rate for GC when using LCI and a high diagnostic performance of ME‐NBI and ME‐BLI for GC compared to those with WLI.

## CONFLICT OF INTEREST STATEMENT

Osamu Dohi received research funding from Fujifilm Co., Ltd. Naohisa Yoshida received research funds and lecture fees from Fujifilm Co., Ltd. The other authors declare no conflict of interest.

## ETHICS STATEMENT

Approval of the research protocol by an Institutional Reviewer Board.

## PATIENT CONSENT STATEMENT

N/A.

## Supporting information


**FIGURE S1** Publication bias funnel plot for studies assessing gastric cancer detection using image‐enhanced endoscopy.

**FIGURE S2** Graphical representation of QUADAS‐2 results.

**TABLE S1** PRISMA 2020 checklist

**TABLE S2** The Cochrane Collaboration tool for randomized trials (RoB2) for assessing the risk of bias among the studies of GC detection.

**TABLE S3** Tabular representation of QUADAS‐2 results.
