# Supplementary figures and images for: Endoscopic detection and diagnosis of gastric cancer using image‐enhanced endoscopy: A systematic review and meta‐analysis
Source: DEN Open. 2024 Aug 13;5(1):e418. doi: 10.1002/deo2.418 (PMC11322228; doi:10.1002/deo2.418)

Supplementary Figure S1

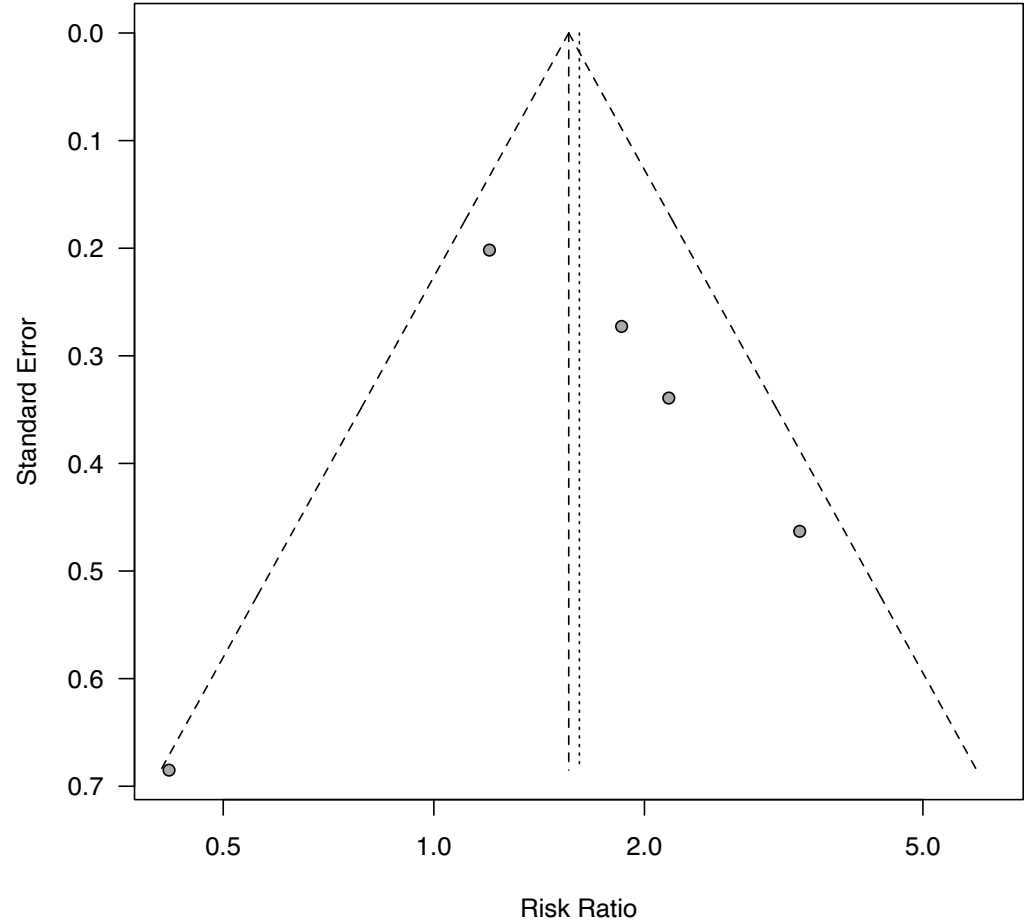

Supplement: Supplementary file 1 — FIGURE S1 Publication bias funnel plot for studies assessing gastric cancer detection using image‐enhanced endoscopy. [file DEO2-5-e418-s004.pdf]

Supplementary Figure S2

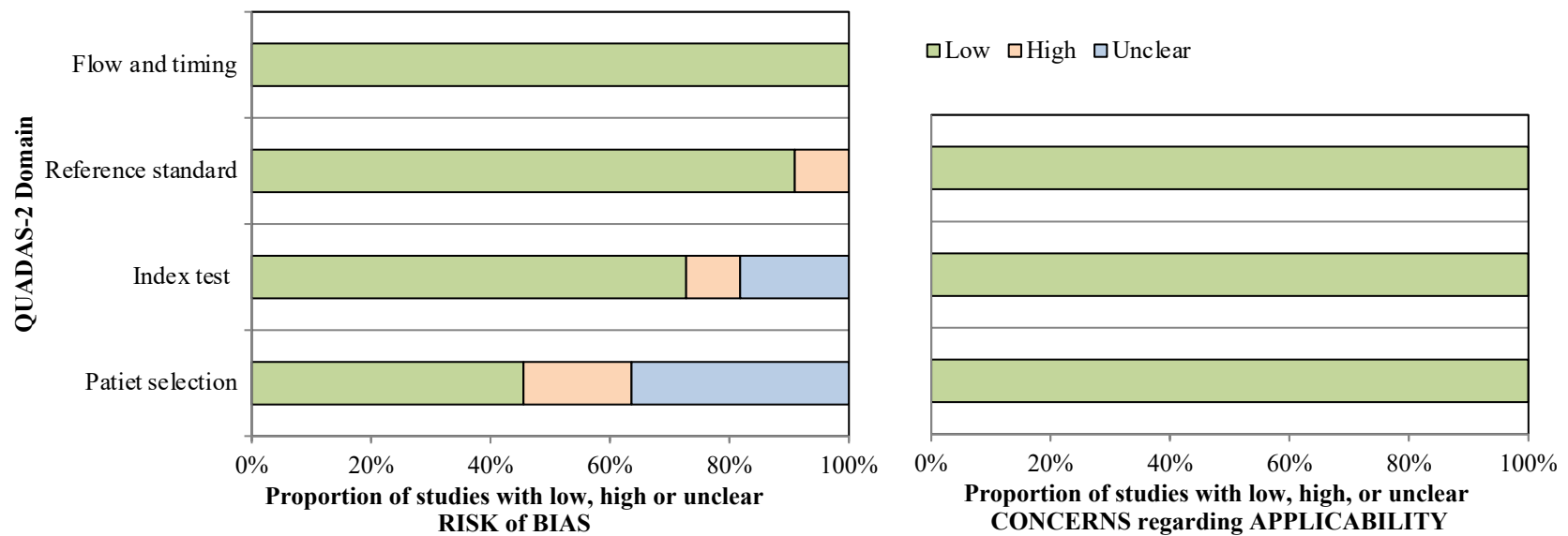

Supplement: Supplementary file 2 — FIGURE S2 Graphical representation of QUADAS‐2 results. [file DEO2-5-e418-s005.pdf]
