# Supplementary material for: Endoscopic detection and diagnosis of gastric cancer using image‐enhanced endoscopy: A systematic review and meta‐analysis
Source: DEN Open. 2024 Aug 13;5(1):e418. doi: 10.1002/deo2.418 (PMC11322228; doi:10.1002/deo2.418)
Supplement: Supplementary file 4 — TABLE S2 The Cochrane Collaboration tool for randomized trials (RoB2) for assessing the risk of bias among the studies of GC detection. [file DEO2-5-e418-s001.docx]

**Supplementary Table S2.** The Cochrane Collaboration tool for randomized trials (RoB2) for assessing the risk of bias among the studies of GC detection.

| Study | Randomization process | Deviation from  intended  interventions | Missing outcome  data | Measurement of the outcome | Selection of reported results | Overall  judgment |
| --- | --- | --- | --- | --- | --- | --- |
| Ang et al.^9^ |  |  |  |  |  |  |
| Dohi et al. ^10^ |  |  |  |  |  |  |
| Yoshida et al.^11^ |  |  |  |  |  |  |
| Ono et al.^12^ |  |  |  |  |  |  |
| Min et al.^13^ |  |  |  |  |  |  |

Low Risk High Risk Some concerns
