# Supplementary material for: Endoscopic detection and diagnosis of gastric cancer using image‐enhanced endoscopy: A systematic review and meta‐analysis
Source: DEN Open. 2024 Aug 13;5(1):e418. doi: 10.1002/deo2.418 (PMC11322228; doi:10.1002/deo2.418)
Supplement: Supplementary file 5 — TABLE S3 Tabular representation of QUADAS‐2 results. [file DEO2-5-e418-s002.docx]

**Supplementary Table S3.** Tabular representation of QUADAS-2 results.

| **Study** | **Risk of bias** | | | |  | **Concerns regarding applicability** | | |
| --- | --- | --- | --- | --- | --- | --- | --- | --- |
|  | Patient selection | Index test | Reference standard | Flow and timing |  | Patient selection | Index test | Reference standard |
| Ezoe et al.^14^ | Low | Low | Low | Low |  | Low | Low | Low |
| Kato et al.^15^ | Unclear | Low | Low | Low |  | Low | Low | Low |
| Ezoe et al.^16^ | Unclear | Low | Low | Low |  | Low | Low | Low |
| Miwa et al.^17^ | Low | Low | Low | Low |  | Low | Low | Low |
| Maki et al.^18^ | Low | High | High | Low |  | Low | Low | Low |
| Tao et al.^19^ | Unclear | Low | Low | Low |  | Low | Low | Low |
| Fujiwara et al.^20^ | High | Unclear | Low | Low |  | Low | Low | Low |
| Nonaka et al.^21^ | Low | Low | Low | Low |  | Low | Low | Low |
| Dohi et al.^22^ | Low | Low | Low | Low |  | Low | Low | Low |
| Kitagawa et al.^23^ | High | Low | Low | Low |  | Low | Low | Low |
| Zhenming et al.^24^ | Unclear | Unclear | Low | Low |  | Low | Low | Low |
